# Pressure- and Temperature-Induced Insertion of N_2_, O_2_ and CH_4_ to Ag-Natrolite

**DOI:** 10.3390/ma13184096

**Published:** 2020-09-15

**Authors:** Donghoon Seoung, Hyeonsu Kim, Pyosang Kim, Yongmoon Lee

**Affiliations:** 1 Department of Earth Systems and Environmental Sciences, Chonnam National University, Gwangju 61186, Korea; dseoung@jnu.ac.kr (D.S.); 197942@jnu.ac.kr (H.K.); 197944@jnu.ac.kr (P.K.); 2 Department of Geological Sciences, Pusan National University, Busan 46241, Korea

**Keywords:** silver-exchanged natrolite, pressure-induced insertion, synchrotron X-ray diffraction, Rietveld refinement

## Abstract

This paper aimed to investigate the structural and chemical changes of Ag-natrolite (Ag_16_Al_16_Si_24_O_80_·16H_2_O, Ag-NAT) in the presence of different pressure transmitting mediums (PTMs), such as N_2_, O_2_ and CH_4_, up to ~8 GPa and 250 °C using in situ synchrotron X-ray powder diffraction and Rietveld refinement. Pressure-induced insertion occurs in two stages in the case of N_2_ and O_2_ runs, as opposed to the CH_4_ run. First changes of the unit cell volume in N_2_, O_2_ and CH_4_ runs are observed at 0.88(5) GPa, 1.05(5) GPa and 1.84(5) GPa with increase of 5.7(1)%, 5.5(1)% and 5.7(1)%, respectively. Subsequent volume changes of Ag-natrolite in the presence of N_2_ and O_2_ appear at 2.15(5) GPa and 5.24(5) GPa with a volume increase of 0.8(1)% and a decrease of 3.0(1)%, respectively. The bulk moduli of the Ag-NAT change from 42(1) to 49(7), from 38(1) to 227(1) and from 49(3) to 79(2) in the case of N_2_, O_2_ and CH_4_ runs, respectively, revealing that the Ag-NAT becomes more incompressible after each insertion of PTM molecules. The shape of the channel window of the Ag-NAT changes from elliptical to more circular after the uptake of N_2_, O_2_ and CH_4_. Overall, the experimental results of Ag-NAT from our previous data and this work establish that the onset pressure exponentially increases with the molecular size. The unit cell volumes of the expanded (or contracted) phases of the Ag-NAT have a linear relationship and limit to maximally expand and contract upon pressure-induced insertion.

## 1. Introduction

Zeolites—one of the most abundant microporous materials—have been widely studied as 3D functional materials and employed as sorbents, catalysts or gas separators due to their various pore sizes and ion-exchange and polar compound adsorption properties [1,2,3,4]. Over the last 60 years, numerous experiments under ambient and applied temperatures have addressed the fundamental behaviors of zeolites and their potential applications in human life and industry. Experiments under pressure conditions, performed in the last few decades, show zeolites exhibit elastic behaviors and pressure-induced anomalous expansion in response to adopted pressure [5]. However, numerous high-pressure studies of natrolite (Na_16_Al_16_Si_24_O_80_·16 H_2_O) have recently been carried out using pressure-induced hydration (PIH) and pressure-induced insertion (PII) accompanying abnormal volume expansion under applied pressures. A notable quantity of studies reported potential for various applications, such as in sequestration of cations and molecules (e.g., Cs^+^, Sr^2+^, Pb^2+^ and CO_2_) and trapping of noble gases (e.g., Ar, Kr and Xe), resulting in the insertion of chemical species by widening the window of natrolite pores using pressure; the noble gases then remain trapped by narrowing the channel opening after pressure release [6,7,8,9,10,11,12,13]. With respect to the crystal structure, the PIH and PII are a consequence of the auxetic behavior of the natrolite framework, which is visualized using a rotating-squares model of framework topology [14]. The natrolite framework is composed of a secondary building unit of T_5_O_10_ (T = Al and Si), which is 3D corner sharing [15]. This unit consists of alternatively bridged Si- and Al-tetrahedra and forms a helical and elliptical channel along the c-axis [16]. The PIH phenomenon was first discovered by Lee et al. when pressurized in water containing PTM [17,18]. The first PIH occurs around 1.0 GPa to form the paranatrolite phase, (Na_16_Al_16_Si_24_O_80_·24 H_2_O) accompanying ~6.7% of unit cell volume expansion and subsequent water insertion with ~3.9% volume contraction occurs at approximately 1.2 GPa to form the super-hydrated natrolite phase (Na_16_Al_16_Si_24_O_80_·32H_2_O). Natrolite shows reversible sequential phase transitions under pressure conditions and is irreversible under simultaneous pressure and temperature conditions. For example, Cs- or Pb-containing natrolite (Cs_16_Al_16_Si_24_O_80_·16H_2_O and Pb_8_Al_16_Si_24_O_80_·16H_2_O, respectively) become pollucite (CsAlSi_2_O_6_·H_2_O) after heating to 160 °C at 2 GPa and lawsonite (Pb_4_Al_8_Si_8_O_28_·4H_2_O) after heating at 200 °C and 4.5 GPa, respectively. The pollucite and lawsonite maintain ~40 wt% of the remaining Cs^+^ and Pb^2+^ cations and show low leaching rates due to tight coordinate bonding with the framework after irreversible phase transition. The pressure- and temperature-driven processes make natrolite a more suitable form for the sequestration of nuclear waste and as long-term storage material under ambient conditions [11,12].

Among the various cation-exchanged natrolites, the silver-exchanged form (Ag-NAT) absorbs water and CO_2_ molecules at comparatively low pressures (0.4(1) GPa and 0.8(1) GPa, respectively), whereas natural natrolite absorbs both at 1.0(1) GPa. The onset pressure of pressure-induced insertion (PII) arises from the circular geometry of the channel window, and we have suggested that one of the possible materials for CO_2_ storage under crustal conditions [9]. We investigated the pressure-induced insertion (PII) of N_2_, O_2_ and CH_4_ gases inside microchannels of the Ag-NAT in order to explore potential material for (ir)reversible gas storage by controlling the pressure and temperature. Herein, we report the structural investigation of Ag-NAT in the presence of N_2_, O_2_ and CH_4_ as PTMs under applied pressure using a Diamond Anvil Cell (DAC).

## 2. Materials and Methods

### 2.1. Sample Preparation

The Ag-NAT was prepared as described by Lee et al. [19]. The starting material, K-NAT, was prepared using a 4 M KNO_3_ (ACS reagent grade from Sigma-Aldrich, St Louis, MO, USA) solution and a ground mineral natrolite (Na_16_Al_16_Si_24_O_80_·16H_2_O, San Juan, Argentina, OBG International) in a 100:1 weight ratio. The mixture was stirred at 80 °C in a reflux system (SciLab Korea Co., LTD, Seoul, Korea) to minimize the loss of water. After 24 h, the solid was separated from the solution by vacuum filtration (SciLab Korea Co., LTD, South). The dried powder was used for the second and third exchange cycles under the same conditions. The final product was washed with deionized water and subsequently air-dried under ambient conditions. Over 99% K-exchange was confirmed by energy-dispersive X-ray spectroscopy (SUPRA25, Zeiss, Germany) (EDS). The Ag-form was prepared with a fully saturated AgNO_3_ solution in the same sequence as above. Stoichiometric analyses of the Ag-NAT on the products were performed using EDS and confirmed that the silver cation was fully exchanged. To determine the amount of H_2_O molecules, Thermogravimetry Analysis (TGA) was performed in the heating range of 25–800 °C at a heating rate of 10 °C/min under a N_2_ atmosphere. The EDS and TGA results are summarized in Table S1 and Figure S1, respectively.

### 2.2. Synchrotron X-ray Powder Diffraction

High-pressure synchrotron X-ray powder diffraction experiments were performed at the 3D and 5A beamlines of the Pohang Accelerator Laboratory (PAL). The primary white beam from the bending magnet at 3D or the superconducting insertion device at 5A, was directed on a Si (111) crystal and sets of parallel slits were used to create monochromatic X-rays with a wavelength of 0.6888(1) Å and 0.6927(1) Å for 3D and 5A, respectively. The diffracted beam was collected using a MAR345 image plate detector (marXperts GmbH, Norderstedt, Germany) as a diffractometer. A LaB_6_ standard (SRM 660c, National Institute of Standards and Technology, USA) was used to calibrate several factors. The calibrated factors were 339.2250(1) mm of sample to detector distance, 1726.827(1) of the X-pixel coordinate of the direct beam, 247.0732(1) of the Y-pixel coordinate of the direct beam, −38.3913(1)° of the rotation angle of the detector and 0.2233(1)° of the tilt angle of the detector.

### 2.3. Diamond Anvil-Cell Preparation

A modified piston–cylinder type DAC (Beijing Scistar Technology CO. LTD., Beijing, China) was used for the high-pressure experiments, equipped with type-I diamond anvils (Almax·easyLab, Ashford, UK) (culet diameter of 700 μm) and tungsten carbide supports. A stainless-steel foil of 250 μm thickness was pre-indented to a thickness of ~100 μm and holes with 300 μm diameter were obtained by electro-spark erosion. The powdered sample of the Ag-NAT was placed in the gasket hole along with some ruby chips for in situ pressure measurements. Ambient pressure data were collected first on the dry powder sample inside the DAC. Subsequently, O_2_ (N_2_ or CH_4_) was added to the sample chamber as a hydrostatic PTM in a cryogenic environment of liquid N_2_ temperature, and then the DAC was sealed at the first pressure point. The pressure of a sample in the DAC was measured by detecting the shift in the R1 emission line of included ruby chips (precision: ±0.05 GPa). The sample was typically equilibrated for approximately 10 min in the DAC at each measured pressure. DAC was heated at 110 °C, 150 °C, 200 °C or 250 °C for 1 h in a dry oven to maintain the hydrostatic pressure around the samples. DAC was then cooled to ambient conditions for 1 h and the pressure was measured again.

### 2.4. Structural Analysis

Pressure- and temperature-dependent changes in the unit cell lengths and volume were derived from a series of whole profile fitting procedures using the GSAS suite of programs in [20]. The background was fixed at selected points and the pseudo-Voigt profile function proposed by Thompson et al. was used to model the observed Bragg peaks, while a March–Dollase function [21] was used to account for the preferred orientation [22]. The structural model at the selected pressure was obtained using Rietveld refinement [20,23]. To reduce the number of parameters, isotropic displacement factors were refined by grouping the framework tetrahedral atoms, framework oxygen atoms and extra-framework species, respectively. Geometric soft-restraints on the T–O (T = Si, Al) and O–O bond distances of the tetrahedra were applied: the distances between Si–O and Al–O were restrained to target values of 1.620 ± 0.001 Å and 1.750 ± 0.001 Å, respectively and the O–O distances to 2.646 ± 0.005 Å for the Si-tetrahedra and 2.858 ± 0.005 Å for the Al-tetrahedra. In the final stage of the refinements, the weights of the restraints on the framework were maintained. Convergence was achieved by simultaneously refining all background and profile parameters, scale factor, lattice constants, two theta zero, preferred orientation function and the atomic positional and thermal displacement parameters. The final refined parameters are summarized in Table S2 and the selected bond distances and angles are listed in Table S3.

## 3. Results and Discussions

**Figure 1:**
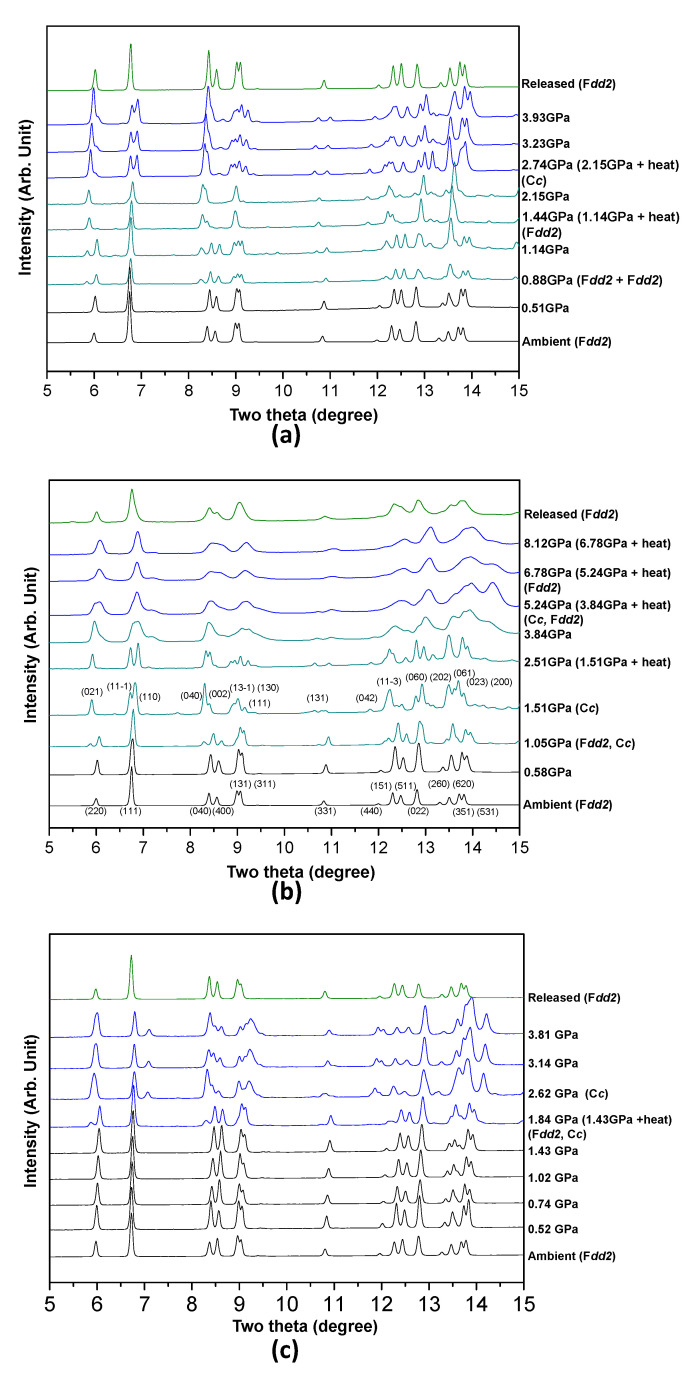
Pressure- and temperature-induced changes of the synchrotron X-ray powder diffraction patterns of Ag-NAT in presence of (**a**) N_2_, (**b**) O_2_ and (**c**) CH_4_. Wavelength of X-ray w 0.6888(1) Å and 0.6927(1) Å in a case of N_2_ (and O_2_) run and CH_4_ run, respectively.

Pressure- and temperature-induced changes in the observed synchrotron X-ray diffraction patterns of the Ag-NAT in the presence of different PTM, N_2_, O_2_ or CH_4_ are shown in Figure 1. The Bragg peaks of the initial phase are indexed to the orthorhombic space group *Fdd2* under ambient conditions [19]. In all cases except the CH_4_ run, the Ag-NAT expands in two stages by applying pressure and temperature. The Bragg peak of (220) is obviously observed to shift to lower two theta angles in all the diffraction data when PII occurs, indicating that structural changes in the *ab*-plane are dominant. The Ag-NAT in the presence of N_2_, the first expanded phase is observed at 0.88(5) GPa owing to the starting pressure-induced insertion (PII) of the N_2_ molecule. The intensity of peaks that belong to the first expanded phase (space group: *Fdd2*) increases up to 1.14(5) GPa, and the ambient phase disappears after heating at 110 °C for an h. The second expanded phase is subsequently observed with a phase transition to monoclinic, *Cc*, at 2.15(5) GPa after heating at 200 °C for 1 h (Figure 1a). The second expanded phase gradually contracts without any further phase change due to pressure and temperature. In the case of O_2_ run, the first expansion of the Ag-NAT is accompanied by a transition to monoclinic, *Cc*, at 1.05(5) GPa. The second expanded phase (orthorhombic: *Fdd2*) was observed at 3.84(5) GPa after heating at 250 °C for 1 h. This phase gradually contracted up to a final pressure of 8.12(5) GPa (Figure 1b). In the case of the CH_4_ run, an expanded phase (space group: *Cc*) is observed at 1.43(5) GPa after heating at 150 °C for 1 h and exists up to 3.81(5) GPa (Figure 1c). In all cases, the Ag-NAT reversibly changes to the initial phase after pressure is released.

**Figure 2:**
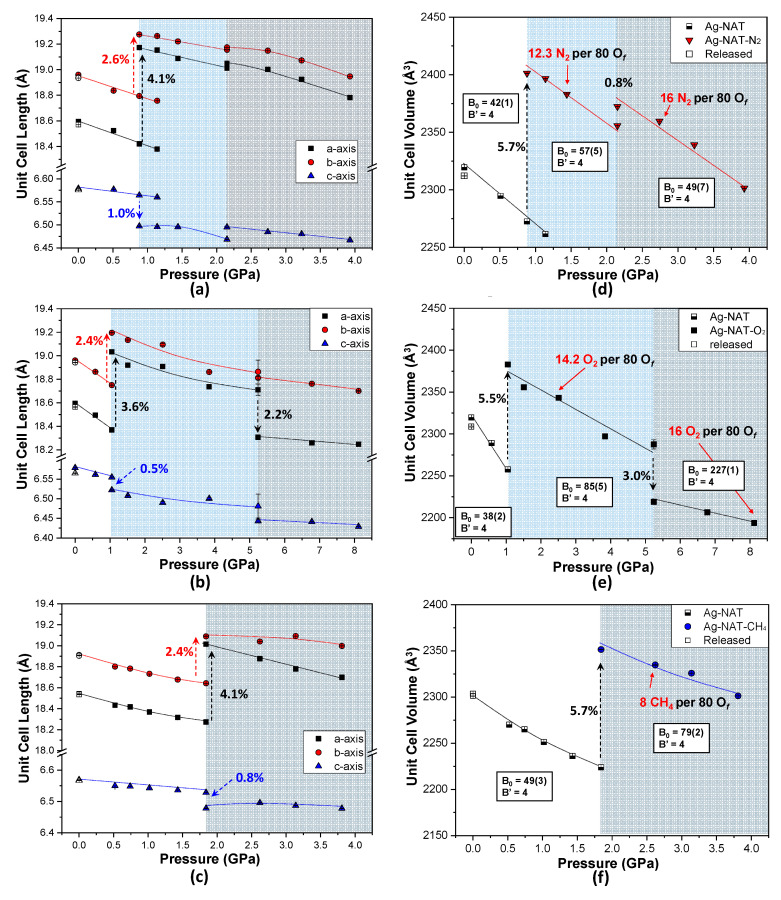
Pressure- and temperature-dependent axial changes of Ag-NAT in presence of (**a**) N_2_, (**b**) O_2_ and (**c**) CH_4_ and changes of unit cell volumes in presence of (**d**) N_2_, (**e**) O_2_ and (**f**) CH_4_. Each open symbol represents volume after pressure released.

A series of whole-profile refinements reveals the details of the compressional changes of the unit cell lengths and volume of the Ag-NAT in the presence of different PTMs (Figure 2). When we convert to a non-conventional *Fd* setting for comparison with the *Fdd2* structure, we find that there are three distinct regions of unit cell parameter changes in the case of N_2_ and O_2_ as PTMs, while there are two regions of unit cell parameter changes in case of the CH_4_ as PTM. In all cases, the *a*- and *b-*axes increase when the first PII occurs (in order of *a*- and *b*-axis, 4.1(1)% and 2.6(1)% at 0.88(5) GPa in the N_2_ run; 3.6(1)% and 2.4(1)% at 1.05(5) GPa in the O_2_ run; 4.1(1)% and 2.4(1)% at 1.84(5) GPa in the CH_4_ run). Compared with the *a*- and *b-*axes, the *c*-axis slightly decreases at the pressure of the first PII (−1.0(1)% in the N_2_ run; −0.5(1)% in the O_2_ run; −0.8(1)% in the CH_4_ run). This phenomenon of axes changing when PII occurs is related to expansion and becoming more circular in the channel window [9]. In the N_2_ run, all axes slightly increase within ~0.5% at 2.15(5) GPa, the onset pressure of the second PII. In regard to O_2_ run, all axes decrease at 5.24(5) GPa. Regarding our Rietveld refinement results and pressure of second PII, the increase of all axes at 2.15(5) GPa in the case of the N_2_ run is dominantly reflected by the PII effect rather than axial contraction by pressure. A decrease in the axes at 5.24(5) GPa and second PII, in the case of O_2_ run compressional effect by pressure is more dominant.

**Figure 3:**
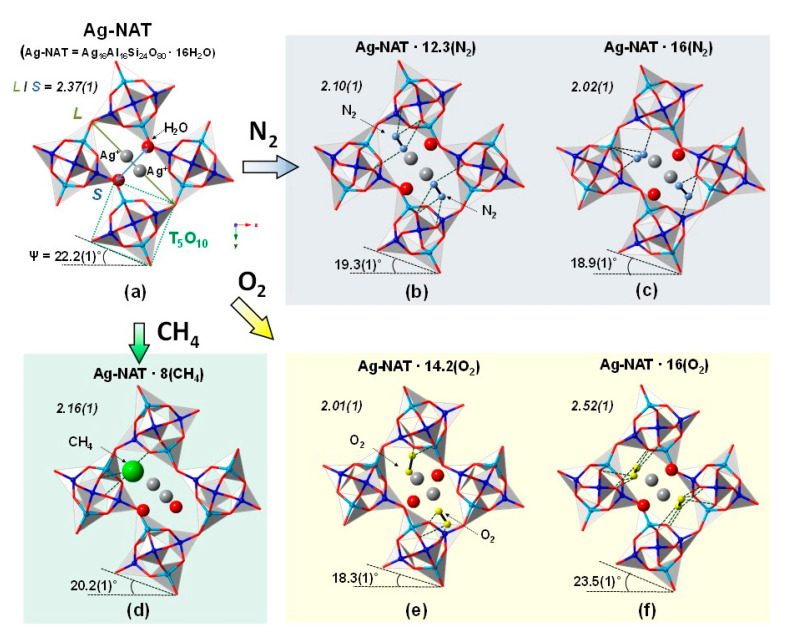
Polyhedral representations of (**a**) Ag-NAT and (**b**) Ag-NAT in N_2_ at 1.44(5) GPa, (**c**) Ag-NAT in N_2_ at 2.74(5) GPa, (**d**) Ag-NAT in CH_4_ at 2.62(5) GPa, (**e**) Ag-NAT in O_2_ at 2.51(5) GPa, (**f**) Ag-NAT in O_2_ at 8.12(5) GPa. Blue balls in tetrahedra illustrate ordered distributions of Al (Si) atoms in the framework. Yellow, dark blue and green balls represent O_2_, N_2_ and CH_4_, respectively. Dashed lines inside channel represent coordinate bonds of gas molecule and framework oxygen.

The unit cell volume of the Ag-NAT with the N_2_ expands 5.7(1)% at 0.88(5) GPa and 0.8(1)% at 2.15(1) GPa, respectively. Except for the abrupt volume expansion at the pressures of the first and second PII (0.88(5) GPa and 2.15(5) GPa, respectively), the unit cell volume linearly decreases with increasing pressure. The volume changes are mainly caused by changes in the *a*- and *b-*axes. Related to our Rietveld refinement results, the number of 12.3 N_2_ molecules per 80 framework oxygen (O_f_) of the Ag-NAT is inserted into the void of the channel at 1.44(5) GPa, after the first PII. At 2.74(5) GPa, 16 N_2_ molecules per 80 of are inserted by the second PII. To understand the relationship between molecules by PII and the compressibility of the Ag-NAT in the presence of N_2_, O_2_ and CH_4_, we used the Birch-Murnaghan equation of state with second order and fixed the derivative of the bulk modulus (B_0_) to 4. In the case of N_2_, the bulk modulus (B_0_) of the Ag-NAT is 42(1) GPa before the first PII occurs at 0.88(5) GPa. Between 0.88(5) GPa and 2.15(5) GPa, the bulk modulus of the Ag-NAT changes to 57(5) GPa. After the second PII occurs at 2.15(5) GPa, the bulk modulus is 49(7) GPa. The bulk modulus changes of the Ag-NAT reveal that the Ag-NAT becomes more incompressible due to insertion of N_2_ molecules into the NAT framework (Figure 2d and Figure 3). In the O_2_ run, the unit cell volume of the Ag-NAT also linearly decreases with pressure except an abrupt expansion of 5.5(1)% at 1.05(5) GPa and contraction of 3.0(1)% at 5.24(5) GPa caused by the first and second PII, respectively. The numbers of 14.2 O_2_ and 16 O_2_ are inserted per 80 of at 2.51(5) GPa and 8.12(5) GPa, after the first and second PII, respectively. The bulk modulus of Ag-NAT in the presence of O_2_ is 38(2) GPa before the first PII at 1.05(5) GPa. From 1.05(5) GPa to 5.24(5) GPa, the bulk modulus of the Ag-NAT increases up to 85(5) GPa. After the second PII at 5.24(1) GPa, the bulk modulus is 227(1) GPa. The highest bulk modulus of 227(1) among our results makes it possible to make fourteen coordinate bonds of O_2_ molecule and framework oxygen (Figure 3f) compared to three to eight bonds are formed after PII in other models (Figure 3a–e). This means that the bonds of O_2_ molecules after the second PII sustain the collapsible framework by pressure and therefore the structure becomes more incompressible. Similar to the case of N_2_ run, the bulk modulus of the Ag-NAT in O_2_ increases after each PII (Figure 2e and Figure 3). In the case of CH_4_, the volume increases 5.7(1)% in response to insertion of 8 CH_4_ per 80 O_f_ at 1.84(5) GPa. Before and after 1.84(5) GPa, the bulk moduli are 55(3) GPa and 79(2) GPa, respectively. After pressure is reduced to ambient pressure, the unit cell volumes of Ag-NAT in all cases recover to a similar volume of volume at ambient pressure (open symbols in Figure 2). Overall, the volume changes are accompanied by the PII and the bulk moduli increase after molecule uptake.

For a detailed understanding of the structural changes before and after pressure-induced insertion of each molecule, Rietveld models were established at selected pressure points in each run (Tables S2 and S3). All models are projected along the [001] direction in Figure 3, and the ambient model is from our previous study [19]. The extra-framework cation (EFC), Ag^+^ and water molecules in the ambient model are located at the center and side of the NAT channel, respectively, and the extra-framework species show an ordered distribution. Silver cations have six-coordinated bonding with four framework oxygens and two water molecules. The geometry of the channel window is determined by measuring the chain rotation angle of the T_5_O_10_ (T = Si and Al) secondary building unit, ψ and the elongation ratio between the lengths of the longest and shortest diagonal (*L/S*) of the eight-membered rings. The lower degree of the chain rotation angle and elongation ratio indicates a more circular shape of the channel window. The ψ value of the Ag-NAT at ambient pressure is 22.2(1)° and the angle decreases to 19.3(1)°, 18.3(1)° and 20.2(1)° after first PII of N_2_, O_2_ and CH_4_ molecules, respectively (Figure 3). In the N_2_ run, the channel window became more circular with increasing amount of N_2_ molecules (12.3 per 80 out of → 16 per 80 O_f_) inside the channel after second PII (19.3(1)° at 1.44(5) GPa → 18.9(1)° at 2.74(5) GPa in Figure 3b,c. The rotation angle of Ag-NAT with O_2_ increases after the second PII (14.2 per 80 O_f_ → 16 per 80; 18.3(1)° at 2.51(5) GPa → 23.5(1)° at 8.12(5) GPa in Figure 3e,f. The changes in the elongation ratio are quite similar to the changes in the rotation angles under pressure. The ratio decreases from 2.37(1) at ambient to 2.10(1), 2.01(1) and 2.16(1) after the first PII of N_2_, O_2_ and CH_4_ molecules, respectively. After the second PII, the ratio decreases to 2.02(1) in the N_2_ run and increases to 2.52(1) in the O_2_ run. Considering a comparatively high pressure of 8.12(5) GPa, the compressional force affects the channel window to become more elliptical rather than becoming circular by insertion and increasing the amount of O_2_ molecules. The difference Fourier map in the channel shows that two unknown sites are adjacent with interatomic distances of ~1.1 Å and ~1.2 Å in all models of Ag-NAT-N_2_ and Ag-NAT-O_2_, respectively. We therefore assign unknown sites to N_2_ and O_2_ and understand that the chemical properties of guest molecules may be retained after inserting into the framework at high pressure.

In all cases, guest molecules were located near the Ag^+^ cation and coordinated with Ag^+^, water molecules and framework oxygen after insertion. The N_2_ molecules are bonded with eight and seven framework oxygens after the first and second PII, respectively, in the case of N_2_ (Figure 3b,c). The six bindings of O_2_ and framework oxygen are formed after the first PII and the number of bonds is changed to fourteen after PII in the case of O_2_ run (Figure 3e,f) due to sustaining the NAT framework at a comparatively high pressure of 8.12(5) GPa in our pressure range. Three framework oxygen atoms are connected by CH_4_ molecules after PII occurs. When CH_4_ is inserted at 2.62(5) GPa, the water molecule migrates to the opposite site of CH_4_ and forms a coordinate bond with the Ag^+^, whereas the atomic positions of the water molecules of the N_2_ and O_2_ models after the first and second PII are similar to those of the ambient model. In the CH_4_ model, CH_4_ molecules may push neighboring host water molecules by repulsion during the CH_4_ occupies one site in the channel and then the water molecule subsequently migrates to the opposite site of CH_4_ to balance the charge distribution inside the channel. Unlike the CH_4_ model, the position of the host water molecules in the pressure models of the Ag-NAT with N_2_ and O_2_ are similar to the position of water molecules in the ambient model because the charge distribution balance is already satisfied due to N_2_ and O_2_ molecules at both sides of the channel. For a detailed understanding of the insertion of different guest molecules inside the Ag-NAT channel, high-pressure spectroscopy experiments were performed.

**Figure 4:**
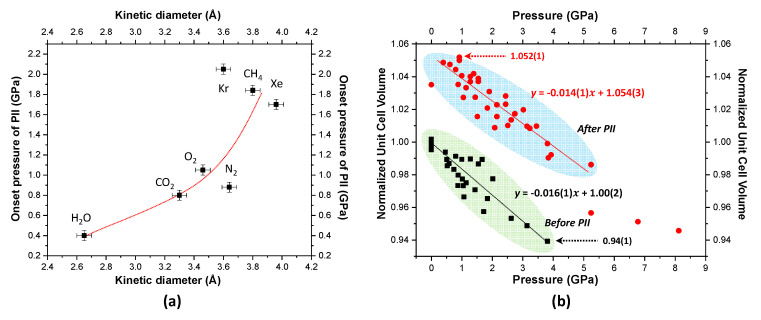
(**a**) Onset pressure of pressure-induced insertion vs. kinetic diameter of molecule as PTM; (**b**) normalized volume plot in a presence of different PTMs (N_2_, O_2_, CH_4_, CO_2_, H_2_O, Xe and Kr) [9,13].

Concomitantly with our previous high-pressure studies of the Ag-NAT, we found that the onset pressure of the first PII exponentially increases as a function of the kinetic diameter of molecules as PTM (Figure 4a) [9,13]. In terms of the kinetic diameter, the water molecule can be most permeable in the angstrom scale channel and therefore, the pressure required for an over-hydrated state is as low as 0.4(1) GPa. In the case of the Xe molecule, however, greater mechanical forces, such as pressure, are required in order to open the channel window of the Ag-NAT and subsequently transfer it into the channel. All our experimental results regarding the unit cell volume of the Ag-NAT obtained using the applied pressure are summarized in Figure 4b. We determined that the Ag-NAT has the limitation of maximum expansion (or contraction) rate of the unit cell volume. Adopting a linear function by the least-square fitting method, the relationship of expanded phases (red symbols) after PII and the as-prepared phases before PII (black symbols) follow Equations (1) and (2), respectively.

y = −0.014(1)*x* + 1.054(3) (1)

y = −0.016(1)*x* + 1.00(2) (2)

where y is the normalized unit cell volume and *x* is the pressure.

The comparison of normalized unit cell volumes shows that their maximum degrees of expansion and contraction are approximately 5% and 6%, respectively, in the pressure range from ambient to 8 GPa. The two values of slope in these functions show that the expanded phase would be more incompressible by the evaluated pressure because the inserted molecules sustain the channel to prevent collapse. These accumulated results can provide guidance for similar experiments using the Ag-NAT in the future. For example, we can expect PII will be almost complete if the volume is increased up to ~5% and follow Equation (1). In other words, the Ag-NAT can absorb more gas if the volume contraction trend follows Equation (2). We expect this approach to also be adapted to other porous materials under non-ambient conditions.

## 4. Conclusions

We established thermo-compressional behaviors of the Ag-NAT in the presence of N_2_, O_2_ and CH_4_ by pressure and temperature conditions with comparative structural changes as the Ag-NAT takes up molecules. The PII occurs twice in the case of N_2_ and O_2_ and once for CH_4_. The unit cell parameters linearly decrease with the evaluated pressure, except when pressure-induced insertion occurs, in which case it abruptly decreases. The pressure required to insert PII into the channel depends on the molecule size, and the incompressibility increases after molecule insertion. We investigated the reversible thermo-compressional interactions of Ag-NAT and guest molecules that are systematically dependent on the ionic diameter of the guest molecules. We expect that our results and experimental approach can be used as fundamental data for application in pressure-induced capture and storage of noble gases or even radioactive species, using microporous materials.

## Supplementary Materials

The following are available online at https://www.mdpi.com/1996-1944/13/18/4096/s1, Figure S1: Graphic result of thermogravimetric analysis of the Ag-NAT. Table S1: Chemical composition of the Ag-NAT calculated from energy dispersive spectroscopy (EDS) method. Table S2: Refined cell parameters and atomic coordinates of Ag-NAT in O_2_, N_2_ and CH_4_ under pressure. Table S3: Selected interatomic distances (Å) and angles (˚) of Ag-NAT in O_2_, N_2_ and CH_4_ under pressure.
