# Supplementary material for: Pressure- and Temperature-Induced Insertion of N2, O2 and CH4 to Ag-Natrolite
Source: Materials (Basel). 2020 Sep 15;13(18):4096. doi: 10.3390/ma13184096 (PMC7560438; doi:10.3390/ma13184096)
Supplement: Supplementary file 1 [file materials-13-04096-s001.pdf]

materials

MDPI

Supplementary Information

Pressure- and Temperature-Induced Insertion of N_2, O_2 and CH_4 to Ag-Natrolite

Donghoon Seoung [1], Hyeonsu Kim [1], Pyosang Kim [1] and Yongmoon Lee [2,*]

[1] Department of Earth Systems and Environmental Sciences, Chonnam National University, Gwangju 61186, Korea; dseoung@jnu.ac.kr (D.S.); 197942@jnu.ac.kr (H.K.); 197944@jnu.ac.kr (P.K.)
[2] Department of Geological Sciences, Pusan National University, Busan 46241, Korea
* Correspondence: lym1229@pusan.ac.kr; Tel.: +82-51-510-2254

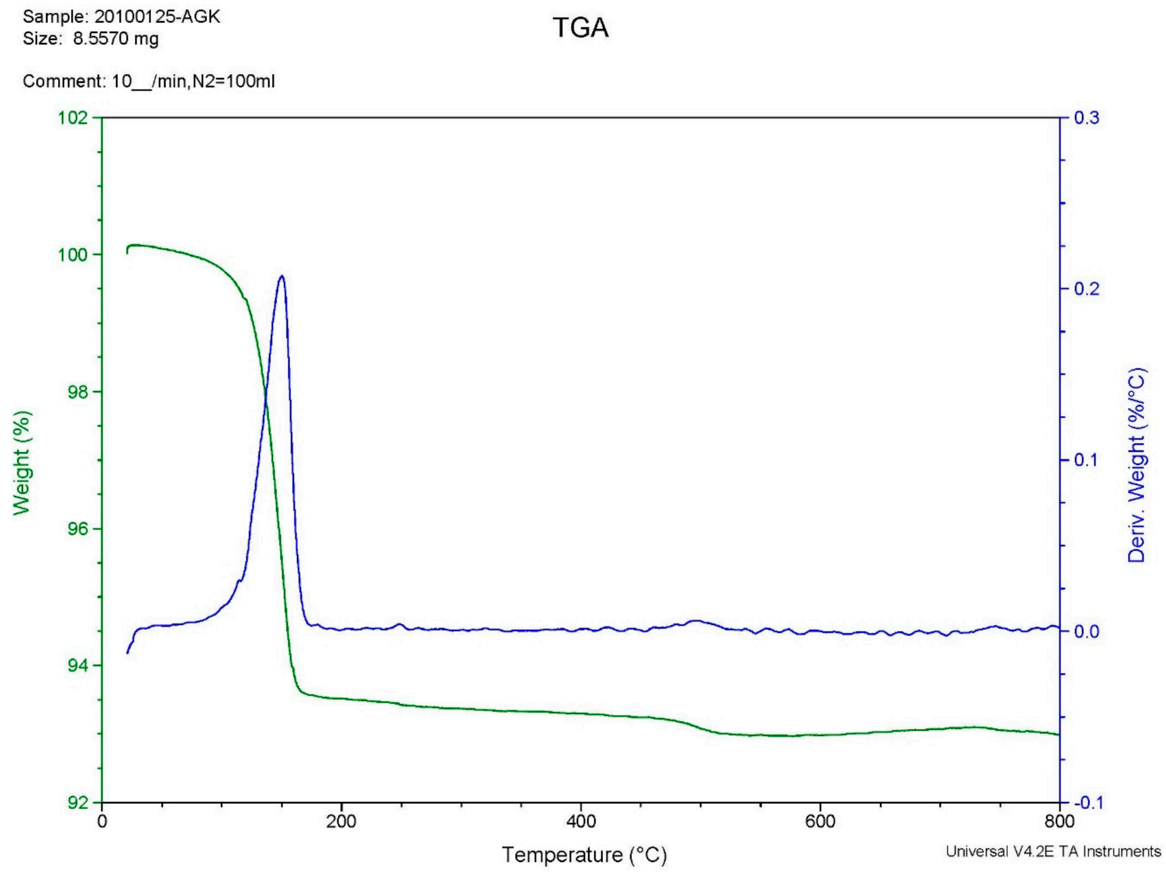

Figure S1. Graphical result of thermogravimetric analysis of the Ag-NAT.

Table S1. Chemical composition of the Ag-NAT calculated from Energy Dispersive Spectroscopy (EDS) method.

Measurement		1	2	3	4	5
Atomic percent (%)	Al	11.70(5)	12.64(5)	12.30(5)	12.24(5)	12.20(5)
	Si	17.05(5)	16.64(5)	17.76(5)	17.81(5)	16.77(5)
	K	0.00(5)	0.00(5)	0.00(5)	0.00(5)	0.00(5)
	Ag	13.05(5)	12.32(5)	12.32(5)	12.31(5)	12.78(5)
	O	58.19(5)	58.40(5)	58.62(5)	58.64(5)	58.24(5)

Table S2. Refined cell parameters and atomic coordinates of Ag-NAT in O_2, N_2 and CH_4 under pressure.[a.]

		Ag-NAT	Ag-NAT + N_2		Ag-NAT + O_2		Ag-NAT + CH_4
Pressure		ambient	1.44GPa (1.14GPa after heat)	2.74GPa (2.47GPa after heat)	2.51GPa (1.51GPa after heat)	8.12GPa (6.78GPa after heat)	2.62GPa
Space group		**F***dd2*	**F***dd2*	**C***c*	**C***c*	**F***dd2*	**C***c*
$_{w}R_{p}$(%), χ^2		4.33, 4.52	3.47, 0.1	4.12, 1.83	3.98, 0.84	4.33, 0.30	4.51, 0.11
Chemical composition		$Ag_{16}Al_{16}Si_{24}O_{80}\cdot 16H_2O$	$Ag_{16}Al_{16}Si_{24}O_{80}\cdot 16H_2O\cdot 12.6N_2$	$Ag_{16}Al_{16}Si_{24}O_{80}\cdot 16H_2O\cdot 16N_2$	$Ag_{16}Al_{16}Si_{24}O_{80}\cdot 16H_2O\cdot 14.2O_2$	$Ag_{16}Al_{16}Si_{24}O_{80}\cdot 16H_2O\cdot 16O_2$	$Ag_{16}Al_{16}Si_{24}O_{80}\cdot 16H_2O\cdot 8CH_4$
Cell parameters (Å)	a	18.5538(1)	19.047(3)	6.4828(7)	6.490(3)	18.33(1)	6.4960(9)
	b	18.9238(1)	19.197(3)	19.144(2)	19.10(1)	18.74(1)	19.040(3)
	c	6.5782(1)	6.492(1)	9.928(1)	9.871(5)	6.443(2)	10.006(2)
	beta	90	90	106.929(6)	106.72(3)	90	109.40(1)
Cell volume (Å^3)	V	2309.64(3)	2373.8(9)	1178.7(3)	1172(1)	2213.0(3)	1167.3(4)
Si(1)	x	0	0	0.454(2)	0.542(6)	0	0.598(2)
8a (**F***dd2*)	y	0	0	0.3862(3)	0.3881(2)	0	0.3671(3)
4a (**C***c*)	z	0	-0.177(3)	-0.148(1)	-0.099(9)	-0.005(2)	-0.197(1)
	U_{iso}^{b}	0.0031(4)	0.007(8)	0.003(4)	0.010(2)	0.001(7)	0.006(5)
Si(2)	x	0.1550(1)	0.1581(1)	0.161(2)	0.246(6)	0.1520(2)	0.336(2)
16b (**F***dd2*)	y	0.2092(1)	0.20989(7)	0.3224(4)	0.3219(3)	0.2170(1)	0.3346(3)
4a(**C***c*)	z	0.6468(3)	0.440(3)	0.013(2)	0.058(9)	0.612(2)	0.018(1)
Si(3)	x			0.495(2)	0.583(6)		0.655(2)
4a (**C***c*)	y			0.0756(4)	0.0751(3)		0.0816(4)
	z			0.146(1)	0.191(9)		0.144(1)
Al(1)	x	0.0390(1)	0.04021(7)	0.869(2)	0.953(6)	0.0344(1)	1.052(2)
16b (**F***dd2*)	y	0.0917(1)	0.0898(1)	0.4604(3)	0.4604(2)	0.0945(2)	0.4620(3)
4a (**C***c*)	z	0.6431(3)	0.433(3)	-0.075(1)	-0.028(9)	0.605(2)	-0.085(1)
Al(2)	x			0.305(2)	0.391(6)		0.456(2)
4a (**C***c*)	y			0.2053(4)	0.2039(3)		0.2187(2)
	z			0.238(1)	0.283(9)		0.252(1)
O(1)	x	0.0285(3)	0.0117(2)	0.403(3)	0.480(6)	0.0031(3)	0.646(2)
16b (**F***dd2*)	y	0.0666(1)	0.06837(4)	0.0332(5)	0.0342(4)	0.07116(6)	0.0290(4)
4a (**C***c*)	z	0.8962(4)	0.681(3)	0.257(1)	0.301(9)	0.853(2)	0.270(1)
	U_{iso}^{b}	0.0026(7)	0.09(2)	0.017(9)	0.03(3)	0.21(2)	0.07(1)
O(2)	x	0.0749(2)	0.0830(1)	0.479(3)	0.570(7)	0.0720(2)	0.545(2)
16b (**F***dd2*)	y	0.1767(2)	0.1704(1)	0.0406(5)	0.0401(4)	0.1804(2)	0.0446(5)
4a (**C***c*)	z	0.6298(8)	0.431(3)	-0.006(1)	0.039(9)	0.602(2)	-0.009(1)
O(3)	x	0.0976(2)	0.0979(1)	0.372(3)	0.465(7)	0.1001(2)	0.519(2)
16b (**F***dd2*)	y	0.0340(2)	0.0251(2)	0.1503(5)	0.1508(4)	0.0321(3)	0.1523(4)
4a (**C***c*)	z	0.5174(6)	0.348(3)	0.117(2)	0.160(9)	0.528(2)	0.150(1)
O(4)	x	0.2080(2)	0.2173(2)	0.098(3)	0.189(6)	0.2106(3)	0.194(2)
16b (**F***dd2*)	y	0.1539(2)	0.1557(1)	0.1696(7)	0.1654(5)	0.1570(2)	0.2045(4)
4a (**C**c)	z	0.7587(6)	0.520(3)	0.296(2)	0.342(9)	0.683(2)	0.259(2)
O(5)	x	0.1826(2)	0.18109(4)	0.239(3)	0.319(6)	0.17723(7)	0.466(2)
16b (**F***dd2*)	y	0.2231(2)	0.2385(2)	0.2892(5)	0.2879(4)	0.2475(3)	0.2996(4)
4a (**C***c*)	z	0.4163(4)	0.216(3)	0.170(2)	0.215(9)	0.388(2)	0.171(1)
O(6)	x			0.023(3)	0.115(6)		0.145(2)
4a (**C***c*)	y			0.2750(5)	0.2746(4)		0.2824(4)
	z			-0.120(2)	-0.076(9)		-0.076(1)
O(7)	x			0.375(3)	0.461(6)		0.503(2)
4a (**C***c*)	y			0.3505(7)	0.3499(5)		0.3501(7)
	z			-0.023(1)	0.024(9)		-0.069(2)
O(8)	x			0.014(3)	0.097(7)		0.229(2)

4a (**C***c*)	y			0.3902(5)	0.3896(4)		0.4079(4)
	z			0.019(2)	0.064(9)		0.045(1)
O(9)	x			0.748(2)	0.835(6)		0.906(2)
4a (**C***c*)	y			0.0912(8)	0.0887(6)		0.1022(7)
	z			0.223(2)	0.273(9)		0.167(1)
O(10)	x			0.596(2)	0.682(6)		0.791(2)
4a (**C***c*)	y			0.4549(5)	0.4567(3)		0.4249(6)
	z			-0.086(2)	-0.033(9)		-0.142(2)
Ag(1a)	x	0.2216(4)	0.2196(7)	0.111(3)	0.18(1)	0.2251(8)	0.751(4)
16b (**F***dd2*)	y	0.0304(4)	0.0305(8)	0.1577(7)	0.158(4)	0.0306(8)	0.3521(8)
4a (**C***c*)	z	0.6483(3)	0.438(5)	-0.087(2)	-0.04(1)	0.581(4)	0.404(2)
	Occu	1.0	1.0	1.0	1.0	1.0	1.0
	U_{iso} [b]	0.0220(2)	0.029(4)	0.051(3)	0.06(1)	0.033(2)	0.028(2)
Ag(1b)	x			0.560(3)	0.66(1)		1.236(3)
4a (**C***c*)	y			0.0960(7)	0.095(3)		0.401(1)
	z			-0.215(2)	-0.174(9)		0.299(3)
	Occu			1.0	1.0		1.0
OW(1a)	x	0.0623(3)	0.048(4)	0.92(1)	0.95(5)	0.060(4)	0.87(1)
16b (**F***dd2*)	y	0.1810(3)	0.163(3)	0.070(5)	0.08(3)	0.176(3)	0.295(5)
4a (**C***c*)	z	0.135(1)	-0.05(2)	-0.023(7)	0.00(3)	0.01(1)	0.216(8)
	Occu	1.0	1.0	1.0	1.0	1.0	1.0
OW(1b)	x			0.78(1)	0.83(7)		0.30(2)
4a (**C***c*)	y			0.272(4)	0.31(3)		0.309(5)
	z			0.280(6)	0.30(3)		0.484(8)
	Occu			1.0	1.0		1.0
N2, O2, CH4[c] (1a)	x		0.190(8)	0.176(9)	0.21(8)	0.149(4)	1.747(9)
16b (**F***dd2*)	y		0.070(6)	0.069(6)	0.07(2)	0.049(5)	0.547(3)
4a (**C***c*)	z		0.01(2)	-0.30(1)	-0.2(1)	-0.117(7)	0.633(6)
	Occu		0.79(6)	1.0	0.9(3)	1.0	1.0
N2, O2 (1b)[d]	x		0.159(6)	0.01(1)	0.19(6)	0.161(5)	
16b (**F***dd2*)	y		0.118(5)	0.084(5)	0.00(4)	0.080(5)	
4a (**C***c*)	z		0.03(2)	-0.356(8)	-0.213(1)	0.046(9)	
N2, O2 (2a)	x			0.09(2)	0.3(1)		
4a (**C***c*)	y			0.306(5)	0.22(2)		
	z			-0.52(1)	-0.42(8)		
	Occu			1.0	0.9(2)		
N2, O2 (2b)[d]	x			0.17(2)	0.24(7)		
4a (**C***c*)	y			0.264(5)	0.27(4)		
	z			-0.44(1)	-0.491(1)		

[a]Esd's are in parentheses. OW denote oxygen site of H_2O molecules.

[b]Isotropic displacement factors (U_{iso}) were refined by grouping the framework tetrahedral atoms, the framework oxygen atoms and the non-framework species, respectively.

[c]CH_4 molecule is assigned by Ne atom based on electron density.

[d]Occupancies of N2(1b), N2(2b), O2(1b), O2(2b) sites are same with these of N2(1a), N2(2a), O2(1a), O2(2a) sites, respectively.

Table S3. Selected interatomic distances (Å) and angles (˚) of Ag-NAT in O_2, N_2 and CH_4 under pressure.[a]

Ag-NAT + N_2				Ag-NAT + O_2				Ag-NAT + CH_4	
1.44GPa (1.14GPa after heat)		2.74GPa (2.47GPa after heat)		2.51GPa (1.51GPa after heat)		8.12GPa (6.78GPa after heat)		2.62GPa	
Space group	**F***dd2*	Space group	**C***c*	Space group	**C***c*	Space group	**F***dd2*	Space group	**C***c*
Si(1) - O(1)	1.6199(3)	Si(1) - O(4)	1.619(1)	Si(1) - O(4)	1.620(1)	Si(1) - O(1)	1.6211(7)	Si(1) - O(4)	1.622(1)
Si(1) - O(5)	1.6196(3)	Si(1) - O(7)	1.619(1)	Si(1) - O(7)	1.620(1)	Si(1) - O(5)	1.6206(7)	Si(1) - O(7)	1.626(1)
mean[b]	1.6198(2)	Si(1) - O(9)	1.620(1)	Si(1) - O(9)	1.620(1)	mean[b]	1.6209(5)	Si(1) - O(9)	1.623(1)
		Si(1) - O(10)	1.620(1)	Si(1) - O(10)	1.620(1)			Si(1) - O(10)	1.621(1)
		mean[b]	1.620(1)	mean[b]	1.620(1)			mean[b]	1.623(1)
Si(2) - O(2)	1.6193(4)	Si(2) - O(5)	1.623(1)	Si(2) - O(5)	1.624(1)	Si(2) - O(2)	1.6204(9)	Si(2) - O(5)	1.625(1)
Si(2) - O(3)	1.6195(4)	Si(2) - O(6)	1.623(1)	Si(2) - O(6)	1.623(1)	Si(2) - O(3)	1.6205(8)	Si(2) - O(6)	1.621(1)
Si(2) - O(4)	1.6194(4)	Si(2) - O(7)	1.622(1)	Si(2) - O(7)	1.623(1)	Si(2) - O(4)	1.6206(8)	Si(2) - O(7)	1.623(1)
Si(2) - O(5)	1.6194(4)	Si(2) - O(8)	1.623(1)	Si(2) - O(8)	1.623(1)	Si(2) - O(5)	1.6200(7)	Si(2) - O(8)	1.621(1)
mean[b]	1.6194(4)	mean[b]	1.623(1)	mean[b]	1.623(1)	mean[b]	1.6204(4)	mean[b]	1.623(1)
		Si(3) - O(1)	1.623(1)	Si(3) - O(1)	1.624(1)			Si(3) - O(1)	1.625(1)
		Si(3) - O(2)	1.623(1)	Si(3) - O(2)	1.624(1)			Si(3) - O(2)	1.625(1)
		Si(3) - O(3)	1.622(1)	Si(3) - O(3)	1.623(1)			Si(3) - O(3)	1.621(1)
		Si(3) - O(9)	1.623(1)	Si(3) - O(9)	1.623(1)			Si(3) - O(9)	1.619(1)
		mean[b]	1.623(1)	mean[b]	1.624(1)			mean[b]	1.623(1)
Al - O(1)	1.7494(4)	Al(1) - O(1)	1.746(1)	Al(1) - O(1)	1.748(1)	Al - O(1)	1.7500(8)	Al(1) - O(1)	1.756(1)
Al - O(2)	1.7495(4)	Al(1) - O(2)	1.747(1)	Al(1) - O(2)	1.747(1)	Al - O(2)	1.751(1)	Al(1) - O(2)	1.753(1)
Al - O(3)	1.7494(4)	Al(1) - O(8)	1.747(1)	Al(1) - O(8)	1.747(1)	Al - O(3)	1.7506(8)	Al(1) - O(8)	1.753(1)
Al - O(4)	1.7493(4)	Al(1) - O(10)	1.747(1)	Al(1) - O(10)	1.747(1)	Al - O(4)	1.7502(8)	Al(1) - O(10)	1.750(1)
mean[b]	1.7494(4)	mean[b]	1.747(1)	mean[b]	1.747(1)	mean[b]	1.7505(4)	mean[b]	1.753(1)
		Al(2) - O(3)	1.747(1)	Al(2) - O(3)	1.747(1)			Al(2) - O(3)	1.754(1)
		Al(2) - O(4)	1.747(1)	Al(2) - O(4)	1.747(1)			Al(2) - O(4)	1.750(1)
		Al(2) - O(5)	1.747(1)	Al(2) - O(5)	1.748(1)			Al(2) - O(5)	1.753(1)
		Al(2) - O(6)	1.747(1)	Al(2) - O(6)	1.747(1)			Al(2) - O(6)	1.755(1)
		mean[b]	1.747(1)	mean[b]	1.747(1)			mean[b]	1.753(1)
Si(1) - O(1) - Al	139.3(1)	Si(3) - O(1) - Al(1)	142.1(6)	Si(3) - O(1) - Al(1)	142.0(4)	Si(1) - O(1) - Al	137.2(2)	Si(3) - O(1) - Al(1)	133.6(5)
Si(2) - O(2) - Al	145.6(2)	Si(3) - O(2) - Al(1)	129.4(4)	Si(3) - O(2) - Al(1)	127.8(3)	Si(2) - O(2) - Al	138.1(3)	Si(3) - O(2) - Al(1)	137.0(4)
Si(2) - O(3) - Al	134.8(2)	Si(3) - O(3) - Al(2)	128.2(5)	Si(3) - O(3) - Al(2)	126.4(4)	Si(2) - O(3) - Al	132.4(3)	Si(3) - O(3) - Al(2)	148.5(5)
Si(2) - O(4) - Al	135.6(1)	Si(1) - O(4) - Al(2)	161.2(7)	Si(1) - O(4) - Al(2)	164.9(5)	Si(2) - O(4) - Al	134.6(2)	Si(1) - O(4) - Al(2)	127.5(5)
Si(1) - O(5) - Si(2)	141.2(1)	Si(2) - O(5) - Al(2)	134.9(5)	Si(2) - O(5) - Al(2)	134.7(4)	Si(1) - O(5) - Si(2)	138.7(2)	Si(2) - O(5) - Al(2)	135.2(4)
		Si(2) - O(6) - Al(2)	130.7(6)	Si(2) - O(6) - Al(2)	129.7(4)			Si(2) - O(6) - Al(2)	136.3(5)
		Si(1) - O(7) - Si(2)	141.9(7)	Si(1) - O(7) - Si(2)	141.8(5)			Si(1) - O(7) - Si(2)	162.0(7)
		Si(2) - O(8) - Al(1)	145.7(6)	Si(2) - O(8) - Al(1)	146.3(4)			Si(2) - O(8) - Al(1)	126.5(5)

		Si(1) - O(9) - Si(3)	155.9(7)	Si(1) - O(9) - Si(3)	157.2(5)			Si(1) - O(9) - Si(3)	131.4(5)
		Si(1) - O(10) - Al(1)	121.7(6)	Si(1) - O(10) - Al(1)	119.2(4)			Si(1) - O(10) - Al(1)	160.4(6)
Average chain rotation angle of T_5O_{10} unit, ψ	19.3(1)°	Average chain rotation angle of T_5O_{10} unit, ψ	18.9(1)°	Average chain rotation angle of T_5O_{10} unit, ψ	18.3(1)°	Average chain rotation angle of T_5O_{10} unit, ψ	23.5(1)°	Average chain rotation angle of T_5O_{10} unit, ψ	20.2(1)°
Ag - O(2)	2.84(3)	Ag(1a) - O(3)	2.23(2)	Ag(1a) - O(1)	2.3(1)	Ag - O(2)	2.60(2)	Ag(1a) - O(2)	2.68(2)
	2.87(3)	Ag(1a) - O(6)	2.31(2)	Ag(1a) - O(5)	3.0(1)		2.54(2)	Ag(1a) - O(3)	2.50(2)
Ag - O(3)	2.39(2)	Ag(1a) - OW(1a)	2.28(9)	Ag(1a) - O(6)	2.27(8)	Ag - O(3)	2.32(2)	Ag(1a) - O(5)	2.65(2)
Ag - O(4)	2.46(2)	Ag(1a) - OW(1b)	2.36(7)			Ag - O(4)	2.47(1)	Ag(1a) - O(6)	2.68(2)
				Ag(1a) - OW(1a)	2.2(5)			Ag(1a) - OW(1a)	2.52(9)
Ag - OW(1)	2.39(8)	Ag(1b) - O(1)	2.66(2)	Ag(1a) - OW(1b)	2.2(4)	Ag - OW(1)	2.72(7)		
	2.87(9)	Ag(1b) - O(2)	2.51(2)	Ag(1a) - O2(1a)	2.7(6)		2.36(8)	Ag(1b) - O(1)	2.50(2)
Ag - N2(1a)	2.64(8)	Ag(1b) - O(5)	2.88(2)			Ag - O2(1a)	2.43(5)	Ag(1b) - O(2)	2.80(4)
		Ag(1b) - O(8)	2.59(2)	Ag(1b) - O(1)	2.69(6)	Ag - O2(1b)	2.94(5)	Ag(1b) - O(5)	2.99(3)
OW(1) - O(1)	2.63(9)	Ag(1b) - OW(1a)	2.60(8)	Ag(1b) - O(2)	2.55(6)			Ag(1b) - O(8)	2.54(3)
OW(1) - O(4)	2.9(1)	Ag(1b) - N2(1a)	2.44(7)	Ag(1b) - O(5)	2.83(5)	OW(1) - O(1)	2.44(6)	Ag(1b) - OW(1a)	3.00(7)
OW(1) - N2(1a)	2.5(2)	Ag(1b) - N2(2a)	2.66(6)	Ag(1b) - O(8)	2.52(7)	OW(1) - O(2)	2.63(7)	Ag(1b) - OW(1b)	2.48(8)
OW(1) - N2(1b)	2.3(1)						2.6(1)		
	3.0(1)	OW(1a) - O(2)	2.97(8)	Ag(1b) - OW(1a)	2.2(3)	OW(1) - O2(1b)	2.60(7)	OW(1a) - O(4)	2.63(9)
		OW(1a) - O(9)	3.00(7)	Ag(1b) - OW(1b)	2.7(3)			OW(1a) - O(5)	2.54(8)
N2(1a) - O(1)	2.6(2)	OW(1a) - O(10)	2.64(9)	Ag(1b) - O2(1a)	2.8(4)	O2(1a) - O(1)	2.71(8)	OW(1a) - OW(1b)	3.0(1)
N2(1a) - O(5)	2.8(2)					O2(1a) - O(3)	2.48(4)		
N2(1a) - N2(1b)	1.103(9)	OW(1b) - O(4)	2.83(7)	OW(1a) - O(2)	2.8(4)	O2(1a) - O(4)	2.65(8)	OW(1b) - O(3)	2.9(1)
		OW(1b) - O(6)	2.32(7)	OW(1a) - O(10)	2.9(4)	O2(1a) - O(5)	2.36(7)	OW(1b) - O(4)	2.9(1)
N2(1b) - O(1)	2.4(1)	OW(1b) - O(7)	3.01(7)			O2(1a) - O2(1b)	1.212(4)	OW(1b) - O(9)	2.4(1)
N2(1b) - O(5)	2.6(1)	OW(1b) - N2(2a)	2.5(1)	OW(1b) - O(6)	2.7(3)				
		OW(1b) - N2(2b)	2.23(9)	OW(1b) - O2(1a)	2.5(5)	O2(1b) - O(1)	2.5(1)	CH4(a) - O(1)	2.89(6)
				OW(1b) - O2(2a)	2.2(7)	O2(1b) - O(4)	2.9(1)	CH4(a) - O(7)	2.88(6)
		N2(1a) - O(1)	2.4(1)	OW(1b) - O2(2b)	2.9(6)	O2(1b) - O(5)	2.3(1)	CH4(a) - O(10)	2.91(7)
		N2(1a) - O(7)	2.9(1)						
		N2(1a) - O(10)	2.7(1)	O2(1a) - O(1)	2.5(5)				
		N2(1a) - N2(1b)	1.11(2)	O2(1a) - O2(1b)	1.2(3)				
		N2(1b) - O(7)	2.06(8)	O2(1b) - Al(1)	2.3(3)				
		N2(1b) - O(10)	2.6(1)	O2(1b) - O(1)	2.0(3)				
		N2(2a) - O(3)	2.4(1)	O2(1b) - O(10)	2.0(4)				
		N2(2b) - O(3)	2.71(8)	O2(2a) - O(4)	2.5(7)				

	N2(2b) - O(7)	2.9(1)	O2(2a) - O(7)	2.5(6)	
			O2(2b) - O(4)	2.6(6)	

[a]Esd's are in parentheses. OW denote oxygen site of water molecules.

[b]Standard deviations computed using $\sigma = 1/n[\sum_{i=1}^{n} \sigma_i{}^2]^{1/2}$.
